# Bilateral Symmetry Detection on the Basis of Scale Invariant Feature Transform

**DOI:** 10.1371/journal.pone.0103561

**Published:** 2014-08-21

**Authors:** Habib Akbar, Khizar Hayat, Nuhman ul Haq, Usama Ijaz Bajwa

**Affiliations:** 1 COMSATS Institute of Information Technology, Abbottabad, Pakistan; 2 College of Arts and Sciences, University of Nizwa, Nizwa, Sultanate of Oman; University of Catania, Italy

## Abstract

The automatic detection of bilateral symmetry is a challenging task in computer vision and pattern recognition. This paper presents an approach for the detection of bilateral symmetry in digital single object images. Our method relies on the extraction of Scale Invariant Feature Transform (SIFT) based feature points, which serves as the basis for the ascertainment of the centroid of the object; the latter being the origin under the Cartesian coordinate system to be converted to the polar coordinate system in order to facilitate the selection symmetric coordinate pairs. This is followed by comparing the gradient magnitude and orientation of the corresponding points to evaluate the amount of symmetry exhibited by each pair of points. The experimental results show that our approach draw the symmetry line accurately, provided that the observed centroid point is true.

## Introduction

Symmetry is an omnipotent phenomenon in real world objects, whether natural or artificial. An object is said to be symmetric, if there is a self-similarity or balance within the object, which can be proved according to the rules of a formal system, i.e. by geometry. Basically there are four types of symmetries in 2D Euclidean geometry [1], namely: bilateral, rotational, translational, and glide-reflectional. Of these, the bilateral symmetry is dominant over all other types of symmetries [1], [2]. Since Symmetry attracts the psychological attention of humans as well as computers [3], it plays an important role in the description and recognition of various objects as well as the understanding of scenes in computer vision and specially robotics [1]. Symmetry has thus an important role in various applications like face analysis [4], indexing of image databases [5], segmentation and detection [6], [7], shape completion [8], detection of vehicles [9], and medical image analysis.

The contribution of this paper is a simple method for detecting bilateral symmetry within medical and real world images by simply comparing the gradient magnitude and orientation of the key points. The key points are obtained by extracting SIFT based features from the input image. The success of the method is primarily dependent on the efficiency of the feature detector, i.e. SIFT. Hence, if SIFT extracts fewer than the required features, in the candidate symmetry area, then the detection rate may be low and the results may suffer. The more the SIFT detect the features, more reliable would be the result. Hence the method requires only robust centroid point of the object. If the detected centroid point conforms the true one, then our approach detects symmetry as ground truth. This validation is done by the manual inspection of the ensued results.

The rest of the paper is structured as follows. Section briefly outlines the related work. The details of our approach are given in Section. The results of our method, when applied to real world images, are elaborated in Section 0.4. Section 0.6 concludes the paper.

## Related Work

The detection of Symmetry has a long history, dating back to 1970's [10] and since then it had got considerable attention of the researchers, in general [11]–[15]. Mancas, Gosselin and Macq [16] detect tumors in medical images, using the bilateral symmetry, by drawing a vertical line called ‘M’ located at the middle of the image. They compute the symmetry simply by subtracting the histogram of the two halves. If the resultant is close to zero, then the two halves are declared symmetric and vice versa. The method is, however, restricted to human body only. Li, Zhang and Kleeman [6] propose a technique to detect bilateral symmetry using the edge pixels for real time applications, like robotic vision. First they convert the image to edge image using Canny's edge detector and then on the basis of edge pixels they detect the line of symmetry. In [7], they extend their own idea to compare the gradient of edge pixels and also detect symmetry in skewed objects. But both the given approaches do not encounter the entire image halves.

Lowe [17] extracts the SIFT feature points and then detect the similarities of the same object in two different images by matching the descriptor vectors of the stored key points with the sensed data. Loy and Eklundh [18] detect bilateral symmetry from features' constellation, by extracting SIFT [17] features to handle bilateral symmetric objects with strong background clutter. Once extracted, each feature has a descriptor vector of 128 elements. They only calculate the mirror descriptor vector for each original one, and then match the original with mirror i.e. cross matching. If the original is match with the mirror and vice versa, then the pair will be symmetric otherwise not. Once symmetric pairs extracted, then on the basis of symmetry magnitude for each pair, they draw a line of symmetry. Liu and Liu [19] extend the idea of [18] in order to detect the curved reflectional symmetry. They first find the symmetric pair points and their middle point, then establish a pair wise consistency graph among all symmetric pair practical's by applying some thresholds values. The line of symmetry is obtained by calculating the Normalize Cross Correlation(NCC) score among the gradients of the original patches in the trapezoid. If the NCC score is above the threshold value, say 0.5, then an edge is imposed among the particles otherwise not. Lee and Liu [20] extend the idea of [18] and detect the curved and curved glide reflectional symmetry. They add the translation component to the reflection. If the translation is zero then the symmetry will be curved reflectional otherwise it will be curved glide reflectional. They extract symmetric pairs and the direction of symmetry line from the orientations of points i.e. pairwise symmetry. The symmetry line is drawn by using the RANdom SAmple Consensus (RANSAC) algorithm in order to fit a curve in 3D Access Parameter Space(APS).

## The Proposed Method

### 0.1 Overview

Our approach is based on the feature points extraction through the SIFT algorithm [21] that extract features, which are invariant with respect to scale, affine transformation (rotation/translation etc.), illumination, 3D view point and noise [17]. On the basis of these feature points, we compute the centroid of the object and draw those in the Cartesian *x*, *y* coordinate system. It is followed by the conversion to the polar coordinate system, in order to facilitate the extraction of symmetric point pairs. In the end the gradient magnitude and orientation of the corresponding symmetric pair points are compared to evaluate the symmetry among the point pairs.

### 0.2 The procedure

For a given candidate line of symmetry, our approach detects symmetry as follows.

1. First some preprocessing is applied to the input image, if required. This may include RGB to intensity image conversion, noise removal and contrast enhancement etc.
2. The resultant intensity image is subjected to SIFT algorithm in order to extract feature points.
3. Ascertain the centroid point of the object of interest by computing the median point of features points, and then subtract the median from all other points in order to find the centriod point. The centroid point is thereby mapped on the origin of the *x*, *y* coordinate system.
4. Convert to polar coordinate system in order to extract symmetric point pairs by applying the basic mathematics rules. In polar coordinate system, each point is represented by 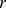 and 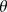, where 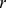 is the distance of the point from the origin and 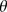 is the angle made with the polar axis. Two points, 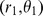 and 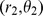, are symmetric with respect to polar axis if 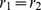 and 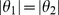. We impose two thresholds, 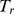 and 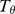, on the differences of 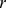-part and 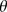-part of corresponding point pairs, respectively, in order to ascertain their symmetry with respect to the polar axis. If both the thresholds are respected, then the points will be symmetric otherwise not.

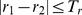



**AND**

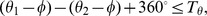
if 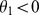 and 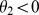,
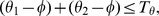
elsewhere.

Where 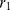, 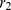 are distances of the key points from the origin and 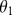, 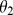 are the respective angles they make with the polar axis, and 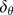 is the degree of increment in order to rotate the line.

1. Once the symmetric point pairs are extracted, the next task is to evaluate the amount of symmetry exhibited by each pair. This is carried out by comparing the gradient magnitude and orientation of the corresponding point pairs with respect to the candidate line of symmetry. The gradient magnitude and orientation are computed via difference pixels from a fixed 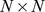 window around each key point, and then convolve the gradient magnitude with a 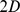 Gaussian function in order to get a weighted magnitude. The gradient magnitude and orientation are accumulated into a histogram - having, say 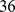 bins each with a period of 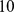. The orientation is then assigned to each key point based on the gradient magnitude, i.e. if the gradient magnitude is greater than, say 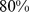, then the corresponding degree will be assigned to that point.

We set two thresholds, namely 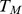 (between 5 and −5) and 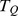 (between 2 and −2), on the gradient magnitude and gradient orientation, respectively and if both thresholds are satisfied according to the following criteria then the points, in a given pair, will be declared symmetric, otherwise not:
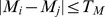



**AND**

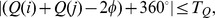
if 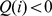 and 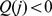,
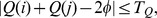
elsewhere.

Where **M** is the gradient magnitude and **Q** is the gradient orientation of the key points.

1. For each point pair, compute the midpoint of the line joining the two points and draw the line joining all such midpoints.

Our approach selects the final line of symmetry among 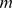 lines, the one which has greater number of symmetric point pairs, i.e. the line against which more symmetric pairs points are detected by our approach. Each pair polls a single vote for symmetry line. A pair will be symmetric if steps 4 and 5 are true, otherwise not.

### 0.3 The candidate lines of symmetry

Our method starts checking the symmetry against the polar axis, i.e. assuming the latter to be the line of symmetry and applying the above procedure to ascertain the symmetry. The candidate line of symmetry is then rotated at fixed increments, 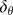, and at each increment the above procedure is repeated. The procedure is stopped when the total increment in rotation reaches 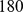 degrees. At the end the list of those lines are maintained against which the symmetry has been ascertained by the above procedure. This procedure allows us to check exactly 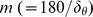 candidate lines for symmetry. A given candidate line makes an angle 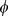 with the polar axis resulted after 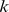 increments, i.e. 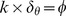, where 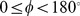.

### 0.4 Statistical measures

In order to summarize the experimental results, we are relying based on the confusion matrix. In the confusion matrix, *true positive* means the symmetry reported by our approach in symmetric object, *false negative* means asymmetry reported by our approach in objects which were actually symmetric. *False positives* mean those asymmetric objects which were wrongly identified by the algorithm as symmetric, while *true negatives* imply asymmetric objects correctly classified as asymmetric.

To identify positive results, *sensitivity* is calculated as under.
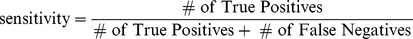
(1)


For identifying negative results, *specificity* is calculated with the following formula.
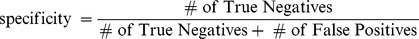
(2)


## Results

### 0.5 Example illustration

**Figure 1:**
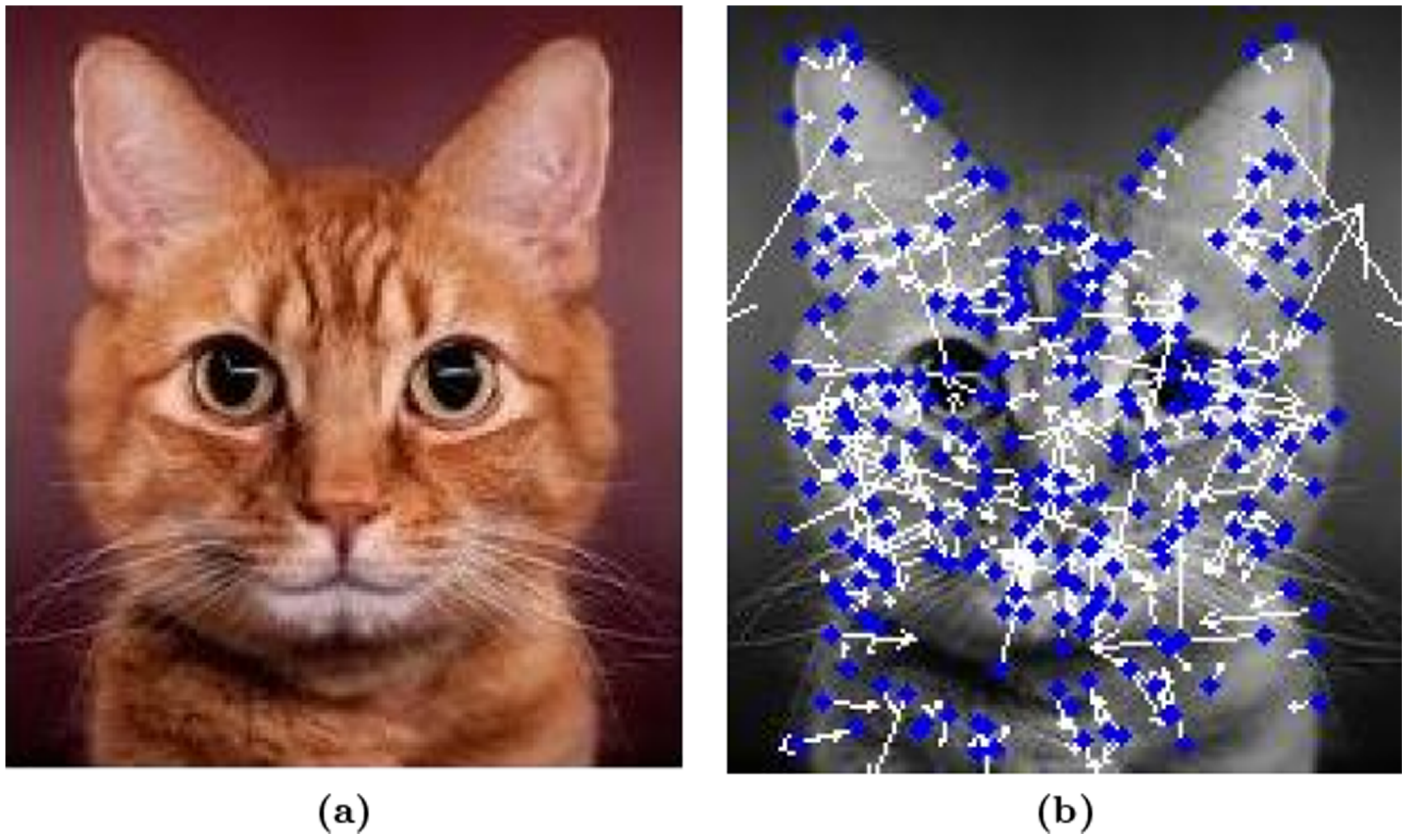
Convert to intensity image and extract the feature points Using SIFT. (a) Original image, (b) After treatment.

As an illustration we are demonstrating the results of our method when applied to the example image, given in Figure 1a. The original image was converted to its intensity image, before subjecting it to SIFT. Then passed the intensity image to SIFT feature extractor to extract feature points, as shown in Figure 1b.

**Figure 2:**
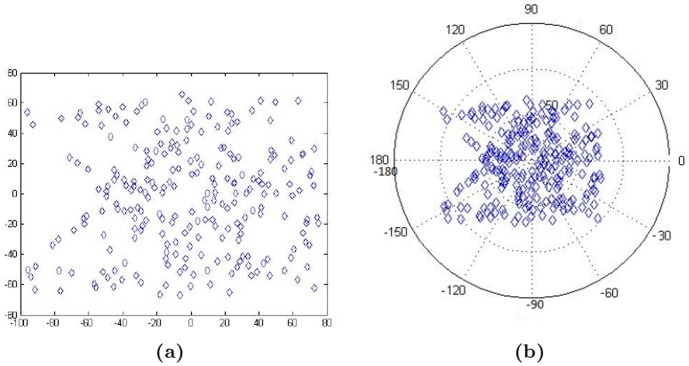
The coordinate systems. (a) Cartesian, (b) Polar.

The next step is to compute the centroid point of the object based on the extracted features. A representation of the points drawn in *x*, *y* coordinate system is shown in Figure 2a. The centroid point is invariant to image rotation with SIFT. Things become easier if one converts to the polar coordinate system in order to extract symmetric pairs points. This may be attributed to the fact that, in polar coordinate system, the rotation of the line is easier to comprehend. In addition, the symmetric pairs are readily identifiable from the feature constellation via the simple symmetry rules in the polar coordinate system, as shown in Figure 2b.

Rather than having the range of 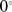 to 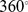, the implementation takes 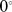 to 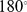 counter clockwise, i.e. positive, and 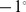 to 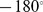 clockwise. For our purpose, we have imposed two small thresholds, 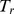 and 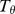 on the distance and angle difference, respectively.

**Figure 3:**
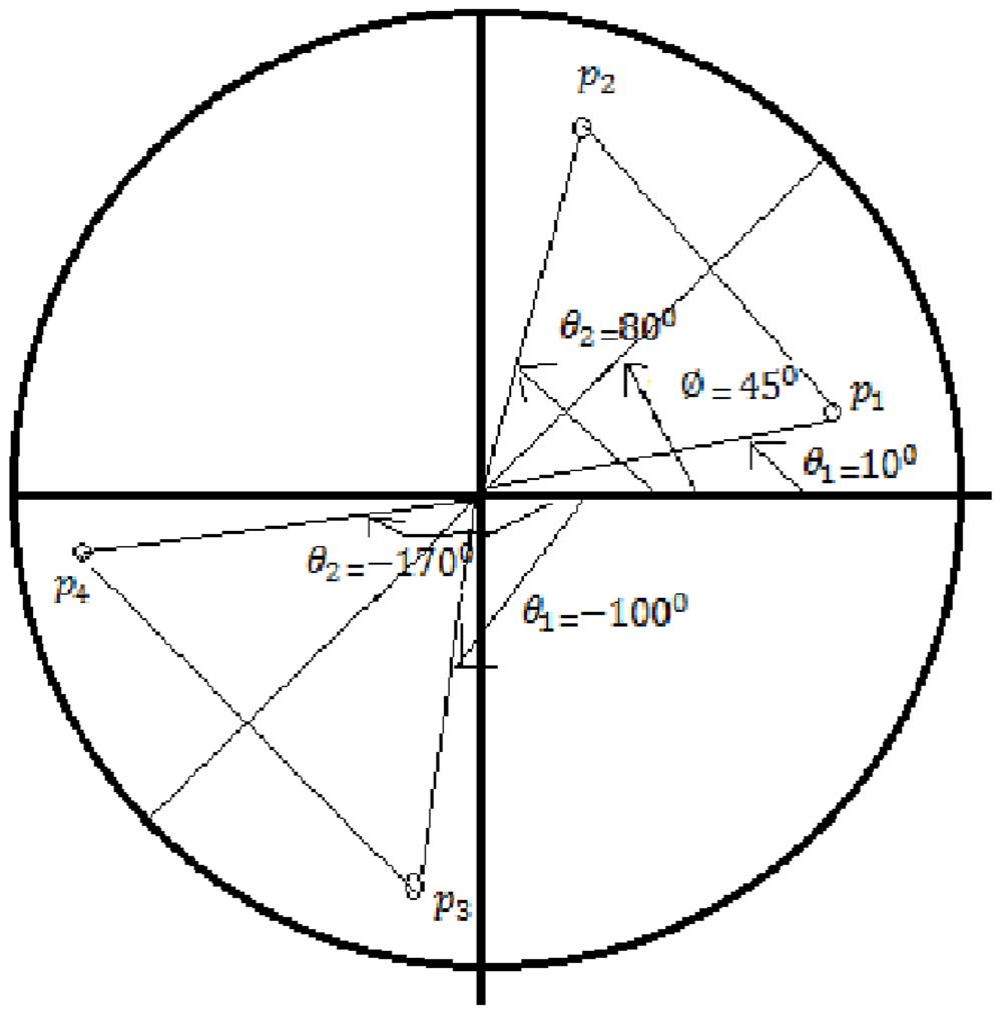
Calculating symmetric points.

Our first candidate line of symmetry is the polar axis, i.e. 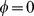, followed by the lines at successive angle increments of 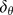; a total of 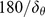 candidates. Against each line, we return point pairs whose *r*-difference and *θ*-difference respect the 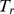 and 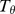 thresholds, respectively. For example, if after 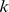 successive increments, the candidate line is at angle (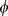) of 45 degrees to the polar axis, our algorithm calculate the symmetric pairs against this line as follows as shown in Figure 3.

Let 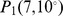 and 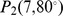 be two points, then angles of both the points are positive, so 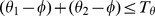 is true, i.e.



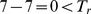
 and
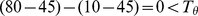



Both the thresholds are respected, so the points are symmetric.

Similarly if 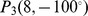 and 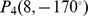 be two points, then angles of both the points are negative, so 
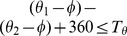
 is true, i.e.



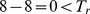
 and
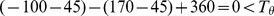



**Figure 4:**
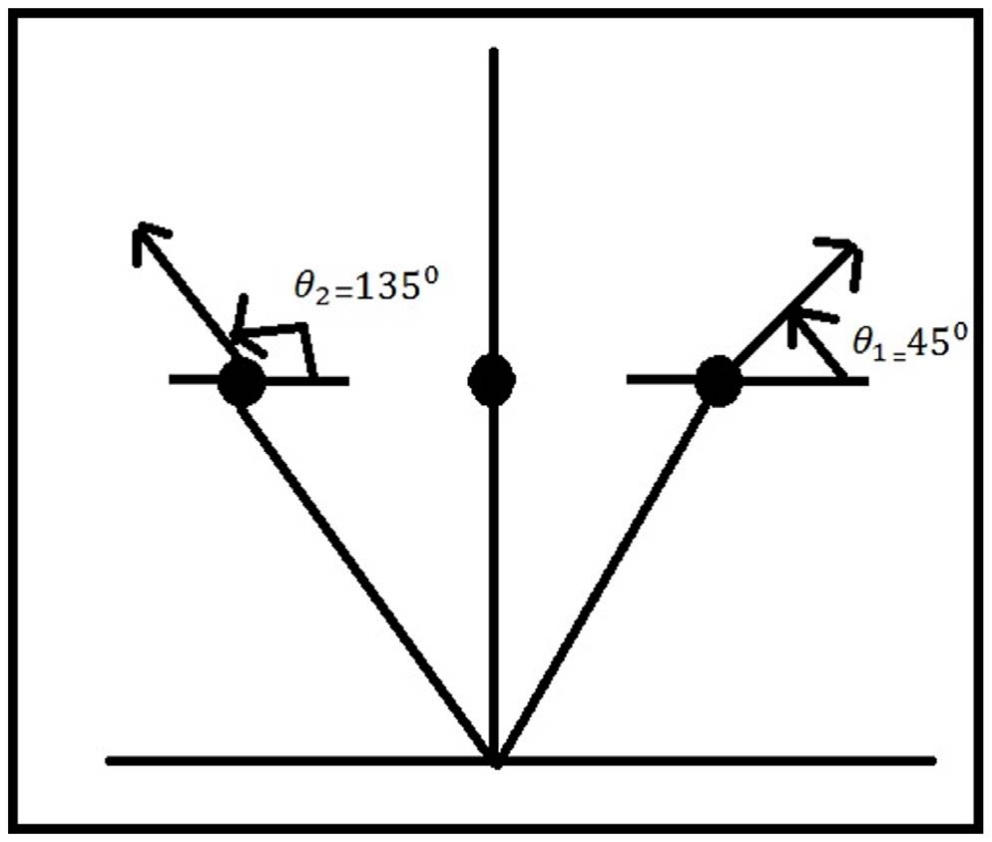
Comparing gradient magnitude and orientation.

Once symmetric pairs are extracted, then we compare the gradient magnitude and orientation of the corresponding points in each pairs. The symmetry of gradient information's is ascertained by comparing with the values of 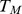 and 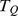 as shown in Figure 4.

Let 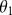 and 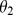 are gradient orientations of two key points, both angles are positive, then 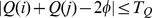 is true, and
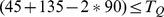



The symmetry measure of each pair is quantified as a function of relative location, gradient magnitude and orientation. If any one of the two condition is false, the pair will not be counted as symmetric.

**Figure 5:**
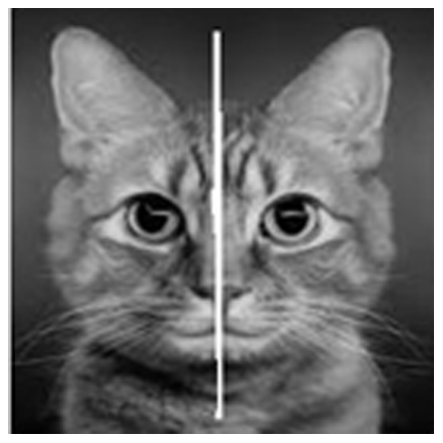
Line of symmetry joining midpoints.

Next midpoints of each point pairs are computed and joined to get the line of symmetry; of all the candidates the one with maximum point pairs is designated as the line of symmetry, as the one shown in Figure 5.

### 0.6 Other Results

**Figure 6:**
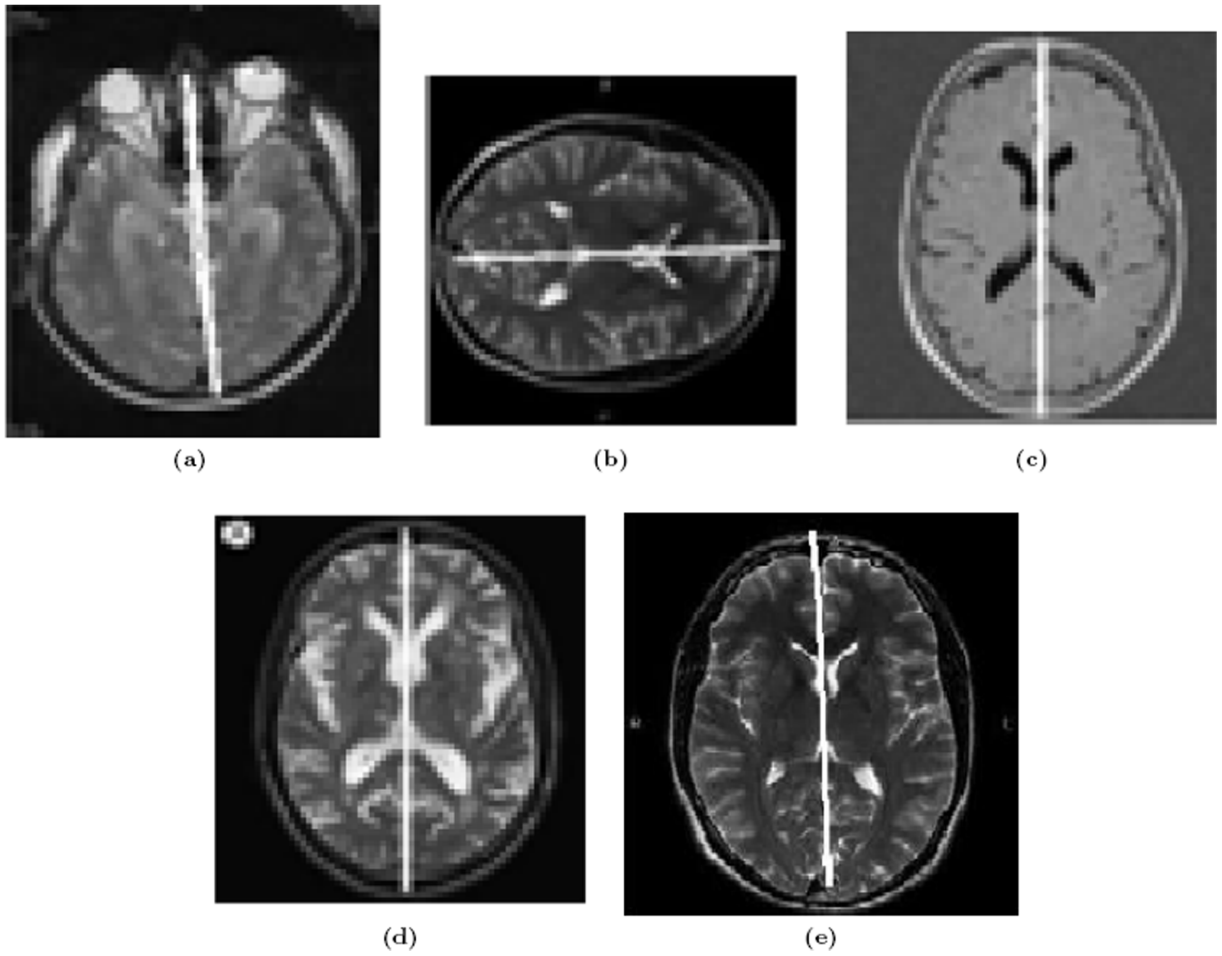
True Positive Results for medical images. (a)–(e) are various examples.

**Figure 7:**
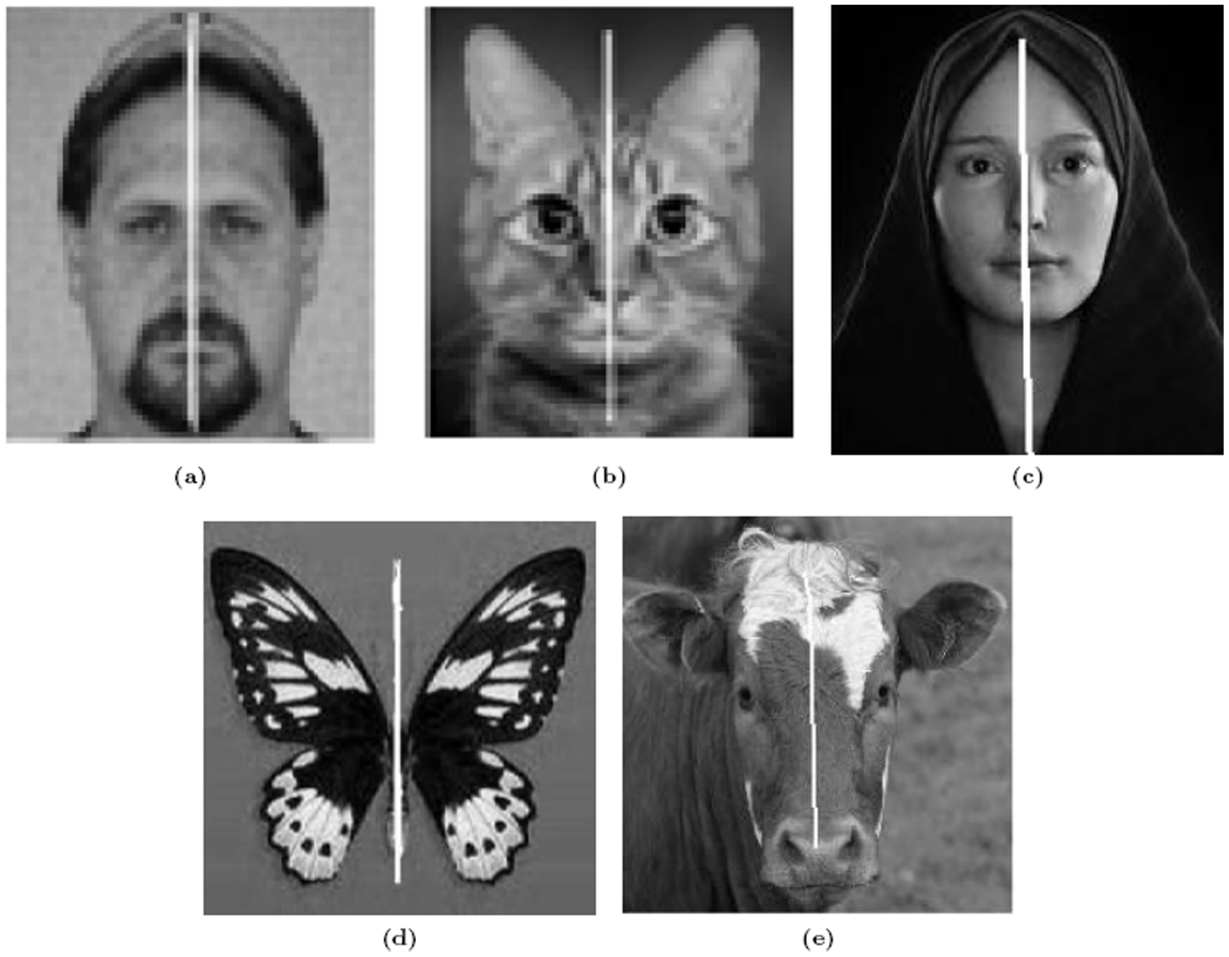
True Positive Results for real-world images. (a)–(e) are various examples.

The feature points are detected using Lowe's SIFT code [17]. On the basis of these feature points, our approach detects bilateral symmetry within medical and real world images as shown in Figure 6 and Figure 7, respectively. We have tested our algorithm on medical and real-world images with strong boundary condition - i.e. frontal images of bilateral symmetric objects - taken from various sources on the Internet. In total, 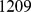 single object images were collected, of which 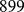 were symmetric images and 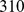 were asymmetric. Out of the former, 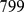 were with no background clutter while 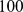 were with background clutter. As can be seen in Table 1, our method identified 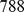 images, out of the 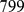 symmetric images (with no background clutter), correctly. This amounts to a sensitivity of 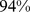. In the case of asymmetric images 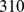 out of 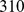 were correctly identified. With frontal symmetric objects, the detection rate of our approach is at par with the method by Loy and Eklundh [18]. For images with background clutter, however, our results are inferior, as evident from Table 2.

**Table 1:** The confusion matrix for experimental dataset.

Test outcome	True Positives	False Negatives
Symmetric	94.47 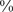	5.53 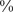
Asymmetric	0 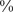	100 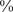
	False Positives	True Negatives

**Table 2:** Comparison of our Approach with Loy's Method.

Test outcome	Our Approach	Loy
Images without background clutter	95.47 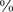	95.47 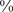
Images with background clutter	60.5 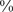	90.9 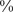

**Figure 8:**
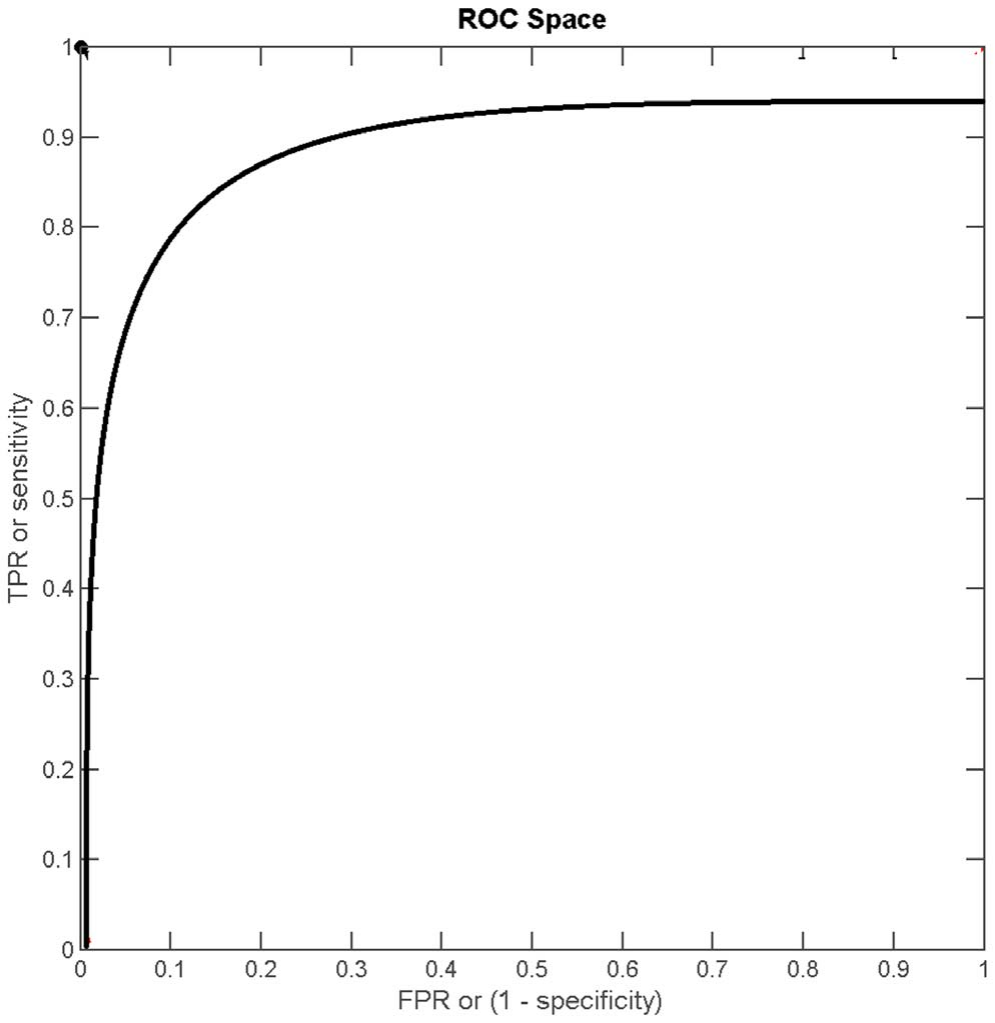
The receiver operating characteristic response.

Figure 8 shows the Receiver Operating Characteristic (ROC) response of our algorithm and its consequential performance, having a curve near to the ideal case. The method has a high area under the curve, being near to 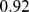, where the maximum is 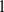. The detection rate for medical images has been found comparatively low, because in such images the symmetry line is not straight; it is a bit curved. Hence our approach detects the symmetry line approximately nearly to the ground truth line. The detection rates of our approach for medical and real world images are shown in Table 3 in the form of a Confusion Matrix. For real world images, the detection rate has been higher, because most real world objects have a straighter line of symmetry.

**Table 3:** The confusion matrix for Medical and real World Images.

Test outcome	True Positives	False Negatives
Real World Images	95.47 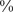	4.53 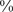
Medical Images	85.1 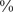	14.9 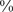

**Figure 9:**
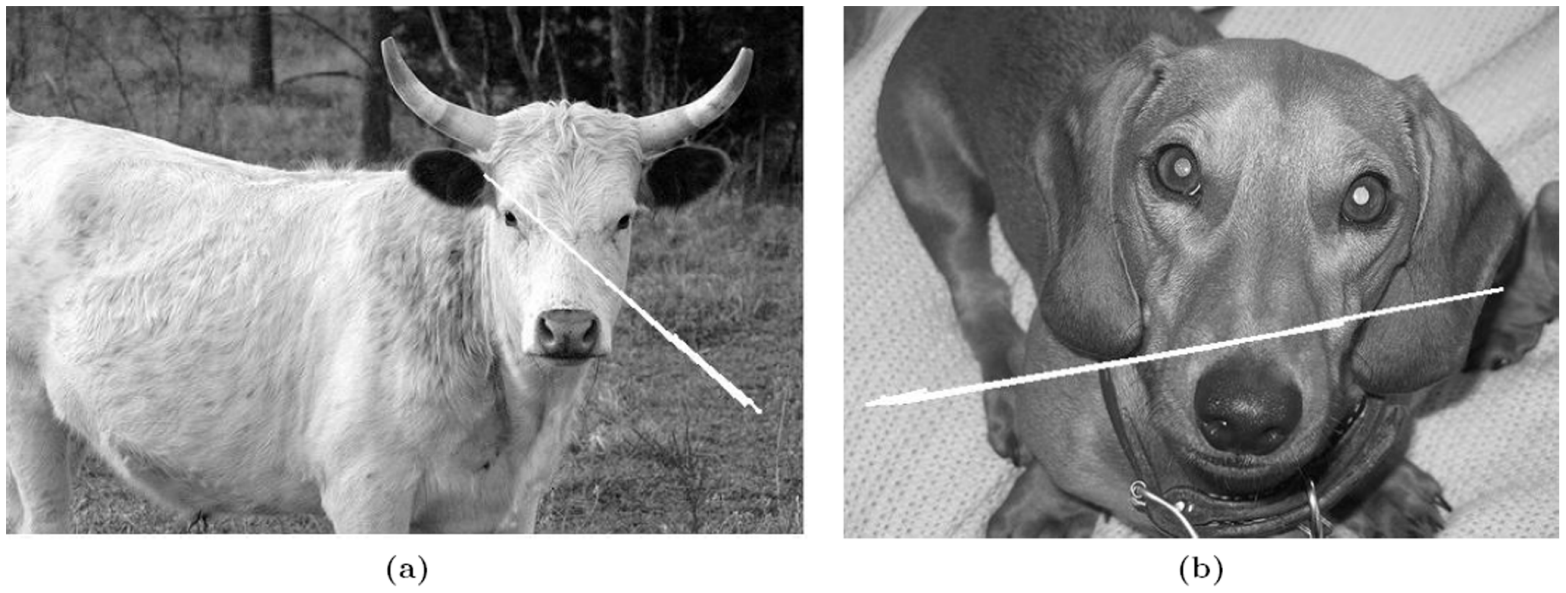
False Negative Results. (a) and (b) are examples.

Two of the example images, where the results are not satisfactory, are shown in Figure 9. Though the contained objects are symmetric, wrong centroids are computed due to too many features in the background. It is to be noted over here that our method would correctly classify symmetric objects only when they appear ‘symmetric’ in the image. In each image of Figure 9, the contained object is by itself symmetric but the way it is photographed, does not give a symmetric view and only the head part constitutes a symmetric object which is unfortunately not detachable from a background that mostly includes the torso. Such kind of ‘localized’ symmetry cases are against our assumption of single-object image.

## Conclusion

The results have been more than satisfactory, provided that the input image is a single object image. With asymmetric objects, the method classified those correctly each time. With symmetric objects too, the outcomes were enviable and only those objects were misclassified which were either partially captured or occluded, or with something in the background. In future we intend to target multiple objects and multiple lines as well as other three aspects of symmetry. Even within asymmetric objects one can identify symmetric parts after some essential pre-processing.
